# Paracetamol, pregnancy and law: What the Texas Tylenol case teaches SA doctors

**DOI:** 10.4102/safp.v68i1.6262

**Published:** 2026-01-19

**Authors:** Suhayfa Bhamjee

**Affiliations:** 1 School of Law, College of Law and Management Studies, University of KwaZulu-Natal, Pietermaritzburg, South Africa

**Keywords:** paracetamol, pregnancy, Texas lawsuit, defensive medicine, ethics, South Africa

## Abstract

**Background:**

Recent litigation in the United States – specifically the Texas Attorney General’s lawsuit against the makers of Tylenol – has reignited global concern over the safety of paracetamol use during pregnancy and its alleged link to neurodevelopmental disorders such as autism and attention deficit hyperactivity disorder (ADHD). Although South African clinical guidelines continue to endorse paracetamol as safe and essential during pregnancy, the legal implications of such international controversies warrant closer scrutiny.

**Methods:**

A narrative legal–ethical review was conducted, drawing on comparative legal frameworks, South African clinical guidelines and recent consensus statements. The article analyses the Texas Tylenol lawsuit, evaluates the evidentiary standards in South African versus US law and considers the ethical obligations of disclosure and risk communication. Sources include peer-reviewed literature, professional guidelines (e.g. Health Professions Council of South Africa [HPCSA] and South African Society of Obstetricians and Gynaecologists [SASOG]) and public health statements. No meta-analysis was performed.

**Results:**

The review found that while South African law requires a causal link for liability, international litigation can influence patient perceptions and clinical behaviour. South African guidelines continue to support paracetamol use in pregnancy, and adherence to these guidelines provides legal and ethical protection. However, practitioners may face increased patient anxiety, pressure to alter prescribing habits and the risk of defensive medicine. Ethical tensions arise between the duty to inform and the risk of fuelling misinformation.

**Conclusion:**

South African family practitioners must remain vigilant in their communication, documentation and reliance on evidence-based consensus.

**Contribution:**

By grounding clinical decisions in local guidelines and ethical reasoning, practitioners can navigate the challenges posed by global controversies while maintaining patient trust and legal defensibility.

## Introduction

Medication safety is a cornerstone of clinical practice, particularly in maternal care. Defensive medicine – where clinicians alter decisions to avoid liability rather than optimise patient outcomes – poses ethical and practical risks. In September 2025, the Texas Attorney General filed a lawsuit against Tylenol manufacturers (Johnson & Johnson and Kenvue), alleging concealed evidence of neurodevelopmental risks.^1^ The lawsuit, amplified by political figures including President Donald Trump and Health Secretary Robert F. Kennedy Jr., has sparked widespread public concern and renewed scrutiny of paracetamol’s safety profile during pregnancy.^2^ This article argues that global litigation, even without direct local effect, influences patient perceptions and clinical behaviour. It explores the legal framework under South African law, ethical principles guiding disclosure and practical recommendations for family practitioners. While most large-scale studies have found no causal link between paracetamol use in pregnancy and neurodevelopment, the legal and reputational consequences of such litigation are far-reaching.^3^

For South African family practitioners, the publicity created by the Texas lawsuit raises critical practical questions: How should clinicians respond when international litigation challenges the safety of a drug widely considered essential in local practice? What are the medico-legal implications of prescribing paracetamol to pregnant patients amid global uncertainty? And how can practitioners balance their ethical duty to inform with the risk of fuelling unnecessary fear?^4^

This article explores these questions through an ethico-legal lens, examining the intersection of clinical practice, public health messaging and medico-legal risk. It argues that while South African guidelines continue to support the use of paracetamol during pregnancy, practitioners must remain vigilant in their communication, documentation and reliance on evidence-based consensus to mitigate potential liability and uphold patient trust.

## Legal context and comparative risk

The Texas lawsuit against the manufacturers of Tylenol is not merely a product liability case – it is emblematic of a broader trend in global health litigation where scientific uncertainty is leveraged to establish legal culpability.^1^ In the United States, the legal system allows for class actions and jury trials in civil claims, often resulting in substantial financial settlements even in the absence of definitive scientific consensus. The lawsuit alleges that Johnson & Johnson and Kenvue failed to warn consumers about the potential neurodevelopmental risks of acetaminophen use during pregnancy,^2^ despite the fact that leading health authorities, including the U.S. Food and Drug Administration (FDA) and the American College of Obstetricians and Gynecologists (ACOG), had not, at the time of instituting litigation, established a causal link between acetaminophen and neurodevelopmental disorders.^3,5^

In contrast, South Africa’s medico-legal framework is more restrained. Product liability claims are governed by the *Consumer Protection Act 68 of 2008*, notably sections 61, 61(4), 61(5) and 115, which collectively require proof of harm and a causal connection to the product in question.^6^ Moreover, the Health Professions Council of South Africa (HPCSA) provides ethical and professional guidelines that, if followed, can serve as a defence in negligence claims.^7^ South African courts generally require a demonstrable causal link between harm and the product in question, and are cautious about claims based on inconclusive scientific evidence, particularly where national clinical guidelines endorse the standard of care.^8^

Nevertheless, the ripple effects of international litigation can influence local practice. South African practitioners may face increased patient anxiety, demands for alternative treatments, or even legal threats based on foreign media coverage. In this context, understanding the legal distinctions between jurisdictions becomes essential. While the Texas case may not have direct legal bearing in South Africa, it underscores the importance of clear documentation, adherence to local clinical guidelines and proactive patient communication.

## Clinical implications for South African family practitioners

Paracetamol remains one of the most commonly recommended medications for managing pain and fever during pregnancy.^4^ Its widespread use is supported by South African clinical guidelines and international consensus, including the joint statement issued by the South African Society of Obstetricians and Gynaecologists (SASOG), the Society of Obstetric Medicine South Africa (SOOMSA) and the South African Society of Ultrasound in Obstetrics and Gynaecology (SASUOG), which affirms its safety when used at therapeutic doses.^4,9^

However, the emergence of high-profile litigation abroad – particularly the Texas lawsuit – has introduced a layer of uncertainty that may affect patient perceptions and clinical decision-making.

Family practitioners are often the first point of contact for pregnant patients seeking reassurance and treatment.

In this context, the implications of international controversy are twofold. Firstly, practitioners may encounter increased patient anxiety, fuelled by social media and global news coverage. Patients may question the safety of paracetamol, demand alternative treatments or express reluctance to follow previously accepted medical advice. Secondly, clinicians may feel pressured to alter prescribing habits or over-explain risks, leading to defensive medicine^10^ and potential under-treatment of conditions such as fever, which itself poses risks to foetal development.^11^

To navigate this landscape, South African family practitioners must rely on clear, evidence-based communication. This includes reaffirming the safety of paracetamol as endorsed by local and international guidelines, and ensure that patient discussions are documented thoroughly, particularly when addressing concerns about drug safety; avoiding speculative commentary on foreign legal cases, while still acknowledging patients’ right to be informed; and ensuring that clinical decisions should be supported by authoritative sources such as the HPCSA’s ethical guidelines and SASOG’s clinical statements.^12^

Ultimately, the goal is to maintain patient trust while safeguarding clinical integrity. By anchoring practice in authoritative local guidelines – such as those issued by the South African Health Products Regulatory Authority (SAHPRA) and SASOG – family practitioners can continue to provide safe, effective care without being unduly influenced by international legal developments.

## Ethics and the duty to inform

The ethical obligations of family practitioners are shaped by the principles of autonomy, beneficence, non-maleficence and justice.^13^ In the context of the paracetamol controversy, these principles intersect in complex ways. Patients have a right to be informed about potential risks associated with treatment,^14^ even when those risks are uncertain or contested.^15^ At the same time, clinicians have a duty to avoid causing unnecessary alarm or harm through the dissemination of speculative or misleading information.

Ethical duties in this context extend beyond legal compliance. Regarding autonomy, patients have a right to informed consent, but clinicians must avoid amplifying unverified risks that could lead to harm. In relation to beneficence and non-maleficence, treating fever during pregnancy prevents teratogenic effects; withholding paracetamol because of fear of litigation may breach these principles. Regarding the principle of justice, equitable care demands that global controversies do not compromise access to safe, evidence-based treatment.

Failure to provide adequate care – underservicing – can attract liability under South African law.^13^ Ethical legitimacy requires balancing transparency with responsibility, guided by HPCSA and SASOG frameworks.

The Texas lawsuit, though legally and geographically distant, has sparked global debate and prompted responses from South African medical bodies, which reaffirmed paracetamol’s safety during pregnancy and cautioned against misinformation.^16,17,18,19^ Should South African practitioners pre-emptively discuss the lawsuit with patients? Is there an ethical obligation to mention unproven risks that have gained media traction? These questions are particularly pressing in maternal care, where anxiety and misinformation can have tangible consequences for both mother and foetus.^20^

Ethically, the duty to inform must be balanced against the duty to protect.^13,15,21,22^ Overemphasis on unverified risks may lead to treatment avoidance, under-medication or substitution with less safe alternatives.^21^ Fever during pregnancy, for example, is a known teratogen, and failure to treat it effectively can result in serious complications.^11^ In this context, withholding paracetamol because of fear of litigation or public pressure may itself constitute a breach of ethical duty.

Professional guidelines, such as those issued by SASOG and the HPCSA, provide a framework for ethical decision-making. These guidelines support the use of paracetamol and encourage practitioners to rely on peer-reviewed evidence and consensus statements when advising patients.^22^ By grounding communication in established science and ethical reasoning, family practitioners can navigate the tension between transparency and reassurance, ensuring that patient care remains both informed and responsible.

## Recommendations for practice

In light of the legal controversy surrounding paracetamol use during pregnancy and its potential impact on patient perceptions, South African family practitioners should adopt a proactive, evidence-based approach to mitigate medico-legal risk and maintain clinical integrity. The following recommendations aim to support practitioners in navigating this complex terrain:

### Anchor clinical decisions in local guidelines

Rely on authoritative sources such as the joint SASOG–SOOMSA–SASUOG statement, which affirms the safety of paracetamol when used appropriately. These guidelines provide a defensible standard of care in both ethical and legal contexts.^4^

### Enhance patient communication

Address patient concerns with empathy and clarity. Acknowledge the existence of international debates without endorsing unproven claims. Emphasise the importance of treating fever and pain during pregnancy and explain the risks of untreated symptoms.^23^

### Document thoroughly

Maintain detailed records of patient interactions, especially when discussing medication risks. Documentation should reflect the rationale for prescribing decisions, reference to clinical guidelines and any patient concerns or refusals.^12^

### Avoid defensive medicine

Resist the urge to alter prescribing practices based on fear of litigation or media pressure. Defensive medicine may compromise patient care and expose practitioners to ethical scrutiny.^10,24^

### Stay informed and educate

Keep abreast of emerging research and legal developments, both locally and internationally. Engage in continuing professional development (CPD) activities that address medico-legal issues and risk communication.^25^

### Collaborate with professional bodies

Seek guidance from medical associations and legal advisors when faced with uncertainty. Collective advocacy and shared resources can help practitioners respond to misinformation and protect the integrity of maternal healthcare.^26,27,28,29^

## Conclusion

The controversy surrounding paracetamol use during pregnancy – sparked by litigation in the USA – offers a cautionary tale about the intersection of law, medicine and public perception. While South African clinical guidelines continue to support the use of paracetamol as safe and necessary, family practitioners must remain vigilant in how they communicate risk, document care and respond to patient concerns. The legal landscape may differ significantly from that of the USA, but the ethical and professional responsibilities remain universal: to provide evidence-based care, protect patient autonomy and guard against the influence of misinformation.

By grounding their practice in local regulatory frameworks and clinical consensus, South African family practitioners can navigate the challenges posed by global controversies with confidence and clarity. In doing so, they not only safeguard their patients’ health but also reinforce the integrity of the medical profession in an era increasingly shaped by legal narratives and public anxiety.
